# The association between benzodiazepine use and sleep quality in residential aged care facilities: a cross-sectional study

**DOI:** 10.1186/s12877-016-0363-6

**Published:** 2016-11-26

**Authors:** Lynna Chen, J. Simon Bell, Renuka Visvanathan, Sarah N. Hilmer, Tina Emery, Leonie Robson, Jessica M. Hughes, Edwin C. K. Tan

**Affiliations:** 1 Faculty of Medicine, Nursing and Health Sciences, Monash University, Melbourne, Australia; 2 Centre for Medicine Use and Safety, Faculty of Pharmacy and Pharmaceutical Sciences, Monash University, 381 Royal Parade, Melbourne, Parkville Victoria 3052 Australia; 3 NHMRC Cognitive Decline Partnership Centre, Hornsby Ku-ring-gai Hospital, Hornsby, Australia; 4 Sansom Institute, School of Pharmacy and Medical Sciences, University of South Australia, Adelaide, Australia; 5 Discipline of Medicine, University of Adelaide, Adelaide, Australia; 6 Aged and Extended Care Services, The Queen Elizabeth Hospital and Adelaide Geriatrics Training and Research with Aged Care (GTRAC) Centre, School of Medicine, University of Adelaide, Adelaide, Australia; 7 Kolling Institute of Medical Research, Departments of Clinical Pharmacology and Aged Care, Royal North Shore Hospital, Sydney Medical School, The University of Sydney, Sydney, Australia; 8 Resthaven Limited, Adelaide, Australia; 9 Aging Research Center, Center for Alzheimer Research, Department of Neurobiology, Care Sciences and Society, Karolinska Institutet and Stockholm University, Stockholm, Sweden

**Keywords:** Sleep, Sleep disorders, Sleep quality, Benzodiazepines, Hypnotics and sedatives, Homes for the aged, Nursing homes

## Abstract

**Background:**

Benzodiazepines are commonly prescribed in residential aged care facilities (RACFs) for their sedative and anxiolytic effects. The objective of this study was to investigate the association between benzodiazepine use and sleep quality in residents of RACFs.

**Methods:**

A cross-sectional study involving 383 participants was conducted in six Australian RACFs. Night-time sleep quality, day-time drowsiness and day-time napping behavior were assessed using a validated questionnaire. Logistic regression was used to compute adjusted odds ratios (AORs) and 95% confidence intervals (CIs) for the association between benzodiazepine use and sleep quality. Covariates included pain, dementia severity, depression, insomnia and other sedative use.

**Results:**

Of the 383 residents (mean age 87.5 years, 77.5% female), 96(25.1%) used a benzodiazepine on a regular basis. Residents who used long-acting benzodiazepines on a regular basis had higher night-time sleep quality than non-users (AOR = 4.00, 95%CI 1.06 – 15.15). Residents who used short-acting benzodiazepines on a PRN only basis had longer daytime napping times than non-users (AOR = 1.77, 95%CI 1.01 – 3.08). No benzodiazepine category was associated with day-time drowsiness.

**Conclusions:**

The association between benzodiazepine use and sleep quality is dependent on the half-life and prescribing pattern of the benzodiazepine. Short-acting PRN benzodiazepines were associated with lower night time sleep quality and longer day-time napping compared to long-acting regular benzodiazepines. Longitudinal studies are needed to determine whether these findings reflect channeling of short-acting agents to residents at higher risk of sleep disorders.

## Background

Ageing is associated with a decrease in sleep length and quality [1]. This is largely attributed to altered circadian rhythms, lowered melatonin levels and increasing rates of medical co-morbidities. Sleep disturbances manifest as decreased total nocturnal sleep time, delayed sleep onset, advanced circadian phase, reduced rapid-eye-movement (REM) sleep and day-time napping and somnolence [2]. Sleep disorders predispose individuals to hypertension, depression, cardiovascular and cerebrovascular disease which further diminish quality of life [3]. In the short term, individuals experience impaired cognition and poor day-to-day functioning. Studies estimate that 50% of individuals over 60 years of age suffer from insomnia, with a higher prevalence amongst women [4].

Residents of RACFs experience a higher prevalence of sleep disorders. This is likely attributable to environmental variables: noise, lighting, temperature and nursing times. It is also attributable to resident characteristics [5]. More than half of residents residing in Australian RACFs have a diagnosis of dementia [6]. People with dementia have higher rates and increased severity of sleep disorders with sleep disturbance present in up to 71% of people with dementia compared to 55.7% of people without dementia [7]. Pain is associated with sleep disorders and is also highly prevalent among residents [8].

The management of sleep problems in residents of RACFs is often multifaceted. Non-pharmacological therapies may include exercise, bright light exposure, nighttime continence care, and cognitive behavioral interventions [9]. However, the results of these interventions are often inconsistent or provide modest benefits on selected aspects of sleep quality. The most common treatment for sleep problems is pharmacological. Benzodiazepines, which enhance the inhibitory effect of gamma-aminobutyric acid (GABA), are commonly prescribed for their sedative and anxiolytic properties [3]. Their sleep-promoting effects make them among the most prevalent medication class among older people [10]. Studies have reported the prevalence of regular benzodiazepine use in Australian RACFs ranging from 12 to 42% [11].

Benzodiazepines are associated with modest improvements to sleep latency and longer sleep duration; however, they suppress deep sleep which compromises sleep’s restorative effects [12]. A meta-analysis by Glass et al. found that the benefits associated with sedative use are marginal and are outweighed by the risk of adverse events. This is particularly clinically relevant for patients at a high risk for falls or cognitive impairment [13]. Older people have increased sensitivity to adverse events, despite growing tolerance to the sedative effects [12]. Adverse events include daytime drowsiness, lethargy, fatigue, agitation, memory loss, impaired coordination and falls [14]. Benzodiazepines may also contribute to cognitive decline in people with dementia [15].

A recent Cochrane review revealed a paucity of appropriately conducted studies that have investigated the benefits and risks of benzodiazepines in people with dementia [16]. As low sleep quality is associated with impaired quality of life [17], it is important to study the effects of benzodiazepine use in vulnerable populations such as people with dementia and those living in RACFs.

The objective of this study was to investigate the association between benzodiazepine use and sleep quality in residents of RACFs.

## Methods

### Design and setting

Data were drawn from a cross-sectional study of permanent residents in six low-level and high-level RACFs in metropolitan Adelaide (population 1.2 million) and regional Mt Gambier (population 25,000), South Australia. In Australia, RACFs predominantly provide support and service to older individuals unable to live independently at home due to frailty, disability or illness. RACFs were classified according to level of support: ranging from aided daily tasks and personal care in low-level RACFs to 24-h nursing in high-level RACFs. The full study protocol has been described previously [18].

### Sample

Permanent residents aged 65 years or older were invited to participate in the study. Inclusion criteria were ability to participate in structured assessments in English. Residents deemed medically unstable (e.g. delirium) or estimated to have less than three months to live by facility staff were excluded from participation.

Of the 664 eligible residents in six RACFs, 603 were invited to participate. Of the 603 residents invited to participate, 220 were excluded (106 declined; 34 were unwell, hospitalized or palliative; 54 had a third party who could not be contacted or did not provide consent; and 26 were excluded for other reasons). The final sample comprised 383 residents. Participants were similar to all residents of the RACFs in terms of age (87.5 years [SD 6.2] vs. 87.3 years [SD 6.4], *p* = 0.66), sex (77.5% female vs. 78.5% female, *p* = 0.90) and dementia diagnosis (44.1% vs. 46.8%, *p* = 0.72).

### Data source

All data were collected by three experienced study nurses who underwent centralized training in the standard administration of the study assessment tools. Data collection took place between April and August 2014.

Demographic, diagnostic and medication data were extracted from each resident’s electronic medical record and medication chart. A standard data extraction form, comprising a series of validated and widely used data collection scales, enabled collection of other clinical data. Both resident self-report and observational scales completed by staff informants were utilized [18]. Where possible, residents chose the most appropriate response through verbal or written communication. If residents were unable, a staff informant with at least two weeks familiarity with the specified resident completed the scale.

### Medication assessment

Data on all charted medications were extracted from each resident’s medication chart. Regular and as-needed (PRN) prescription, non-prescription and complementary and alternative medications were considered. Regular use was defined as a documented regular sequence of administration in the resident’s chart.

Medications were coded according to the Anatomical Therapeutic Chemical (ATC) classification system, as recommended by the World Health Organization. Benzodiazepines were defined as anxiolytic benzodiazepine derivatives (N05BA), hypnotic and sedative benzodiazepine derivatives (N05CD) or benzodiazepine related drugs (N05CF). A resident was considered a benzodiazepine user if he or she was charted a benzodiazepine on a regular or PRN basis. Benzodiazepines were classified according to half-life into two categories: short-acting (half-life ≤24 h) and long-acting (half-life >24 h). Temazepam, oxazepam, alprazolam, bromazepam, triazolam, midazolam, lorazepam, zopiclone and zolpidem were classified as short-acting benzodiazepines. Diazepam, clobazam, nitrazepam, flunitrazepam were classified as long-acting benzodiazepines. Non-benzodiazepine sedative drugs included other sedatives and hypnotics (e.g. melatonin, antihistamines) and medications with prominent sedative side effects (e.g. antipsychotics, antidepressants, antiepileptics, opioid analgesics).

### Sleep quality

The primary outcome measure was sleep quality assessed using a previously validated six-item questionnaire [19]. The questionnaire comprised three domains: night-time sleep quality (overall self-reported sleep quality, sleeping duration, restfulness), day-time drowsiness and day-time napping behavior (frequency and duration) (Table 2). The six-item questionnaire has been used previously among older people living in self- and assisted-care villages to assess the association between sleep quality and falls [20].

The scoring of the individual sleep domains within the questionnaire was adapted from the method described by St George et al [20]. The night-time sleep quality score was derived from an aggregate of the three night-time sleep items, with equal weighting for each domain. This yielded a night-time sleep quality score ranging between 3 and 15. Higher night-time sleep quality was operationally defined as a score of 3–10 and lower sleep quality as a score of 11–15, based on median score. Day-time drowsiness was dichotomized as drowsy or non-drowsy. Average number of hours napping per week was calculated from the questions pertaining to napping frequency and duration. Residents were categorized as napping <3.5 h per week (less than half an hour per day), or ≥3.5 h per week [20].

### Covariates

*Co-morbidities* were extracted from resident electronic medical records and included depression, insomnia and history of falls. The Charlson Comorbidity Index (CCI) was calculated for each resident [21]. *Dementia severity* was measured in residents with and without a dementia diagnosis with the Dementia Severity Rating Scale [22]. Resident *agitation and aggression* was assessed using the Neuropsychiatric Inventory Nursing Home version (NPI-NH) [23]. *Activities of daily living* were assessed with the Katz Activities of Daily Living (ADL) index [24]. *Pain* was observed and assessed using the Pain Assessment in Advanced Dementia (PAINAD) Scale [25].

### Statistical analysis

Descriptive analyses were conducted using Pearson Chi-square tests for categorical variables, Student’s t-tests for continuous variables and Mann-Whitney U tests for ordinal variables. Odds ratios (ORs) and 95% confidence intervals (CIs) of the associations between different benzodiazepine categories and sleep domains were computed with binary logistic regression analyses. Analyses were adjusted for age, sex, dementia severity rating scale, any pain reported on the PAINAD scale, depression, insomnia and non-benzodiazepine sedative use. These factors were used to adjust the analyses because they were deemed clinically relevant based on previous research [2]. Sensitivity analysis was performed by initially removing insomnia diagnosis to assess for potential over-correction, and this did not affect the results. Benzodiazepine use was analyzed separately based on half-life classification (short or long-acting) and type of prescription (regular or PRN). Separate models were run for the association between each benzodiazepine category and each different sleep quality domain i.e. night-time sleep quality, day-time drowsiness and day-time napping behavior. The reference groups were those residents not taking these drugs.

Missing values were imputed with multiple imputation with five iterations. This was because multiple imputation is superior to complete case analyses when data are missing at random [26]. Values were imputed for sleep quality questionnaire (24.8% missing) and PAINAD (1.0% missing). Analyses were undertaken using the Statistical Package for the Social Sciences (SPSS, version 21.0, Chicago, IL, USA).

### Ethical considerations

The study was approved by the Royal Australian College of General Practitioners (RACGP) National Research and Evaluation Ethics Committee and the Monash University Human Research Ethics Committee. All participants were informed about the study verbally and in writing. Written informed consent was obtained from the residents, or their guardian, next of kin, or significant other if unable. All study procedures were conducted in accordance with the Australian National Statement for Ethical Conduct in Human Research.

## Results

Of the residents, 229 (59.8%) were able to self-complete the sleep questionnaire, with the remainder completed by a staff informant or carer. Demographic characteristics of residents in RACFs with lower and higher night-time sleep quality were similar (Table 1). The mean age of residents was 87.5 years (SD = 6.19). Of the 383 participants, 297 (77.5%) were female and 135 (35.2%) lived in high-level RACFs. In total, 169 (44.1%) had a dementia diagnosis and 230 (60.1%) had a depression diagnosis. Residents with lower night-time sleep quality were more likely to exhibit pain (34.6% vs 25.0%, *p* = 0.04) compared to those with higher night-time sleep quality (Table 1).

**Table 1:** Characteristics of residents with higher and lower sleep quality in Australian residential aged care facilities

	All residents	Higher sleep quality (NTSQS >10)		Lower sleep quality (NTSQS ≤10)		*P* value
	383	202	53%	181	47%	
*Demographic characteristics*						
Age (mean ± SD)	87.53 (6.19)	87.62	(6.16)	87.43	(6.22)	0.77^a^
Female	297	160	79.2%	137	75.7%	0.39^b^
High Level Care	135	79	39.1%	56	30.9%	0.09^b^
Born in Australia	298	150	74.3%	148	81.8%	0.10^b^
*Functional characteristics*						
Charlson’s comorbidity index (median, IQR)	2.00 (1.00, 3.00)	2.00	(1.00, 3.00)	2.00	(1.00, 4.00)	0.39^c^
ADLs (median, IQR)	1.00 (4.00, 6.00)	3.00	(1.00, 6.00)	4.00	(1.00, 6.00)	0.66^c^
DSRS (mean ± SD)	19.09 (16.87)	20.19	(16.57)	17.85	(17.10)	0.21^a^
PAINAD, any	113	50	24.8%	63	34.8%	0.04^b^
*Medical diagnoses and medications*						
Dementia	169	94	46.5%	75	41.4%	0.35^b^
Insomnia	48	22	10.9%	26	14.4%	0.37^b^
Anxiety	146	78	38.6%	68	37.6%	0.81^b^
Depression	230	115	56.9%	115	63.5%	0.21^b^
Any non-benzodiazepine sedatives	272	145	71.8%	127	70.2%	0.71^b^

*NTSQS* night-time sleep quality score, *ACAT* aged care assessment team, *ADLs* activities of daily living, *DSRS* dementia severity rating scale, *PAINAD* Pain Assessment in Advanced Dementia Scale. *SD* standard deviation, *IQR* interquartile range

a.Student’s *t*-test

b.Pearson’s Chi-square test

c.Mann-Whitney *U* test

Overall, 96 (25.1%) residents were charted regular benzodiazepines and 121 (31.6%) were charted PRN benzodiazepines. Eighty residents (20.9%) used benzodiazepines on a PRN only basis, with the majority being short-acting formulations (n = 69). Forty-one (10.7%) residents used more than one category of benzodiazepine.

Residents who used benzodiazepines had a higher prevalence of diagnosed insomnia (21.3% vs 4.9%, *p* < 0.01), anxiety (44.9% vs 32.2%, *p* = 0.01) and depression (68.0% vs 53.2%, *p* < 0.01) than residents who did not use benzodiazepines. Residents taking benzodiazepines had higher prevalence of agitation or aggression compared to those residents not taking benzodiazepines (34.3% vs 19.5%, *p* < 0.01).

Resident responses to the six item sleep questionnaire are shown in Table 2. There were no statistically significant differences between users and non-users of benzodiazepines in any of the sleep questionnaire items or domains.

**Table 2:** Sleep quality in users and non-users of benzodiazepines in Australian residential aged care facilities. (Based on 6-item questionnaire and derived sleep domain scores)

	All residents (*n* = 383)	Non-Benzodiazepine user (*n* = 204)		Benzodiazepine user(*n* = 179)		*P*
Q1: SLEEP QUALITY: Over the past month rate the quality of your night time sleep?						
Very poor	10	3	1.5%	7	3.9%	0.22^a^
Poor	44	19	9.3%	25	14.0%	
Okay	96	56	27.5%	40	22.3%	
Good	162	84	41.2%	78	43.6%	
Very good	72	43	21.1%	29	16.2%	
Q2: SLEEP DURATION: How much sleep do you get each night?						
< 4h	7	3	1.5%	4	2.2%	0.06^a^
4-6h	89	43	21.1%	46	25.7%	
6-8h	180	93	45.6%	87	48.6%	
6-10h	84	49	24.0%	35	19.6%	
> 10h	22	15	7.4%	7	3.9%	
Q3: RESTFUL SLEEP: How restless or restful is your sleep as a rule?						
Very restless	10	4	2.0%	6	3.4%	0.16^a^
Restless	62	30	14.7%	32	17.9%	
Not very	60	29	14.2%	31	17.3%	
Restful	191	107	52.5%	84	46.9%	
Very restful	60	34	16.7%	26	14.5%	
Q4: WAKING CONDITION: How clear headed do you feel when you wake in the morning?						
Alert	184	104	51.0%	80	44.7%	0.58^a^
Slightly drowsy	151	71	34.8%	80	44.7%	
Fairly drowsy	33	22	10.8%	11	6.1%	
Very drowsy	15	8	3.9%	7	3.9%	
Q5: NAPPING FREQUENCY: How regularly do you sleep, nap, or lie down during the day?						
Nearly every day	171	87	42.6%	84	46.9%	0.47^a^
A few times a week	52	30	14.7%	22	12.3%	
Once or twice a week	47	21	10.3%	26	14.5%	
Once or twice a month	27	21	10.3%	6	3.4%	
Never or hardly ever	86	45	22.1%	41	22.9%	
Q6: NAPPING DURATION: In general how much sleep would you say you get on these days?						0.52^a^
None	73	39	19.1%	34	19.0%	
Less than 1/2h	109	54	26.5%	55	30.7%	
1/2 to 1h	129	70	34.3%	59	33.0%	
1 to 2h	54	32	15.7%	22	12.3%	
More than 2h	18	9	4.4%	9	5.0%	
SLEEP QUALITY DOMAINS						
Night-time Sleep Quality Score (Category)						
Higher	202	113	55.4%	89	49.7%	0.26^a^
Lower	181	91	44.4%	90	50.6%	
Night-time Sleep Quality Score (Mean)	10.29 (2.45)	10.52	(2.37)	10.02	(2.52)	0.05^b^
Day-time Drowsiness (Category)						
Alert	183	103	50.5%	80	44.7%	0.26^c^
Drowsy	200	101	49.5%	99	55.3%	
Napping Score (Category)						
< 3.5h/week	256	137	67.2%	119	66.5%	0.82^a^
≥ 3.5h/week	127	67	32.8%	60	33.5%	
Napping Score (Mean)	3.02	3.01		3.03		0.97^b^

a.Mann-Whitney U test

b.Student’s *t*-test

c.Pearson’s chi-square tes

### Night-time sleep quality

Overall, 181 (47.3%) residents had lower night-time sleep quality (score ≤10). In adjusted analyses, use of regular long-acting benzodiazepines was significantly associated with higher sleep quality (AOR = 4.00, 95%CI = 1.06 – 15.15) (Table 3). Use of PRN short-acting benzodiazepines was associated with lower night-time sleep quality, however this did not reach statistical significance (AOR = 0.59, 95%CI = 0.34 – 1.02).

**Table 3:** Association between benzodiazepine use and sleep quality, day-time drowsiness and napping in Australian residential aged care facilities

		Association between BZD and higher night-time sleep quality			Association between BZD and day-time drowsiness			Association between BZD and longer average napping time (≥3.5 h per week)		
	*N*	AOR^a^	CI	*P*	AOR^a^	CI	*P*	AOR^a^	CI	*P*
Any BZD	179	0.82	0.53 – 1.26	0.36	1.27	0.81 – 1.98	0.30	1.04	0.65 – 1.66	0.87
Any Regular										
- short-acting	83	0.91	0.54 – 1.53	0.71	1.05	0.62 – 1.80	0.85	0.70	0.40 – 1.22	0.21
- long-acting	14	4.00	1.06 – 15.15	0.04	1.53	0.48 – 4.90	0.48	0.32	0.07 – 1.50	0.15
PRN only										
- short-acting	69	0.59	0.34 – 1.02	0.06	1.29	0.73 – 2.26	0.38	1.77	1.01 – 3.08	0.04
- long-acting	11	0.77	0.22 – 2.67	0.68	1.29	0.37 – 4.46	0.69	1.18	0.30 – 4.70	0.81

*AOR* adjusted odds ratio, *CI* 95% confidence interval, *BZD* benzodiazepine

^a^each model adjusted for age, sex, *DSRS* (Dementia Severity Rating Scale), *PAINAD* (Pain Assessment in Advanced Dementia Scale), depression, insomnia and use of non-benzodiazepine sedatives

### Day-time drowsiness

Of benzodiazepine users, 49.5% reported day-time drowsiness compared to 55.3% of non-users. No statistically significant associations were found in unadjusted and adjusted analyses between use of any benzodiazepine category and day-time drowsiness (Table 3).

### Average weekly napping time

There was no significant difference in mean daytime napping time between benzodiazepine users and non-users overall (3.03 vs 3.01 h per week, *p* = 0.97) (Table 1). However, the use of PRN only benzodiazepines was found to be associated with increased day-time napping time (AOR = 1.77, 95%CI = 1.01-3.08).

## Discussion

This was one of the first studies to investigate the association between benzodiazepines and sleep quality in RACFs [27, 28]. The primary finding was that PRN only short-acting benzodiazepine use was associated with lower night-time sleep quality while regular long-acting benzodiazepine use was associated with higher night-time sleep quality.

The prevalence of benzodiazepine use amongst RACF residents in our study was similar to previous Australian and international studies [11, 29, 30]. The proportion of residents classified as having lower night-time sleep quality is similar to previous research that reports between 40-65% of older adults have some form of sleep disturbance [31]. St George et al., using the same six-item questionnaire, found a similar prevalence of lower night-time sleep quality and an association between pain and lower night-time sleep quality. Contrary to previous studies, our study found no significant association between night-time sleep quality and age, sex and medical diagnoses, in particular dementia severity and depression [2, 4, 10]. Both age and dementia are known to adversely impact on circadian rhythms, with increasing dementia severity impacting further on sleep.

There was a statistically significant positive association between regular long-acting benzodiazepine use and higher night-time sleep quality. However, the sample size for regular long-acting benzodiazepines (n = 14) was modest in comparison to other categories. Traditionally, long-acting benzodiazepines such as diazepam are more commonly prescribed to manage anxiety disorders [32]. As such, residents using long-acting benzodiazepines may not have had existing sleep problems.

In our study, there was an association between use of short-acting benzodiazepines on a PRN basis only and lower night-time sleep quality, although this was not statistically significant. The effects of benzodiazepine use on sleep quality have been mixed. For example, a systematic review of randomized controlled trials found that sedative hypnotics offer statistically significant, but small, improvements in sleep quality [13]. However, long-term benzodiazepine use is associated with tolerance which may negate the therapeutic effects of benzodiazepines [27]. As our study was cross-sectional we were not able to investigate tolerance and duration of benzodiazepine use. A cross-sectional study by Béland et al. also reported an association between benzodiazepines and poor sleep quality, which the authors attributed to the physiological tolerance mechanism [33]. The use of short-acting benzodiazepines, such as temazepam, is recommended as a first line short-term treatment for insomnia by the Australian Therapeutics Guidelines [32]. Thus, this finding may be attributed to the role of using short-acting benzodiazepines, as required, in residents who are experiencing poor sleep quality. Individuals with poor sleep quality may be more likely to be prescribed PRN short-acting benzodiazepines, and this may highlight potential confounding by indication.

There was an association between PRN benzodiazepine only use and longer day-time napping. Australian Therapeutic Guidelines recommend the use of PRN medication in both the treatment of insomnia and anxiety [32]. Historically, short-acting benzodiazepines have been attributed with lower rates of adverse events and lowered risk of residual next-day sequelae such as napping [34]. Clinician awareness of preventing adverse events is seen through prescribing habits and patterns. Thus, our findings may reflect channeling of PRN medications to residents susceptible to daytime napping, particularly those with acute rather than chronic insomnia [35]. This study found no association between benzodiazepine use and day-time drowsiness, whereas previous research indicates benzodiazepine users are at a greater risk of somnolence [10, 28]. The different effects of varying formulations and regimens of benzodiazepines on the sleep domains highlights the need for informed decision making when it comes to prescribing these medications.

### Strengths and limitations

Demographic and diagnostic characteristics were similar among participants and non-participants, thus the study sample presented is likely representative of all residents in the RACFs. Despite having a larger sample size than previous research [27], generalizability of the study results may be limited and findings may not be applicable to other demographics and settings. The cross-sectional study design limited our ability to infer causality between associated factors.

Strengths lie in our method of data collection. Diagnostic data were extracted from each resident’s most recent electronic medical records. Likewise, medication use was extracted from current medication charts, providing a more accurate reflection of actual medication exposure than relying on prescribing or dispensing data to ascertain medication use. We conducted separate analyses for regular and PRN benzodiazepine use and for short and long-acting benzodiazepine use. However, we did not investigate the possible impact of benzodiazepine dose and duration, which could have effects through tolerance with long term use, or withdrawal worsening sleep quality in residents with recent dose reduction or recent change from long to short acting benzodiazepines. Consideration was made for a range of clinically relevant covariates; however, we did not adjust our analyses for sleep disorders other than insomnia, such as obstructive sleep apnea and restless leg syndrome [10]. Other potential factors previously studied, such as environment, poor sleep hygiene, social connections and fitness levels, were not considered [10, 20]. The majority of observational or self-reported data in our study were gathered with widely-used, previously validated scales that have been demonstrated to be appropriate for use in aged care facilities or people with dementia. However, the results should be interpreted in light of the fact that 40.2% of the sampled residents needed assistance with completing the sleep questionnaire, and a proportion of data were imputed. Residents needing assistance with the questionnaire were largely cognitively impaired. Although a staff or carer proxy was used in these cases, they had to be familiar with the resident and their sleep behavior (i.e. had cared for the resident for a minimum of two weeks). By including residents with cognitive impairment, generalizability within the aged care setting was maximized. The small number of regular long-acting benzodiazepine users, and hence wide confidence interval, means these results should be interpreted with caution.

## Conclusions

The association between benzodiazepine use and sleep quality in residents of aged care facilities is dependent on the half-life and prescribing pattern of the benzodiazepine. Short-acting PRN benzodiazepines were associated with lower night time sleep quality and longer day-time napping compared to long-acting regular benzodiazepines. Longitudinal studies are needed to determine whether these findings reflect channeling of the short-acting agents and PRN prescribing to residents at higher risk of sleep disorders, or acute rather than chronic insomnia. Further research should be undertaken to explore the effect of benzodiazepine dose and duration on sleep quality.

## Abbreviations

AOR: Adjusted odds ratio
ATC: Anatomical Therapeutic Classification
CI: Confidence interval
GABA: Gamma-Aminobutyric acid
OR: Odds ratio
PRN: Pro re nata
RACF: Residential aged care facility
RACGP: Royal Australian College of General Practitioners
REM: Rapid eye movement
